# The Causes of Congenital Club-foot, with Remarks upon the Time for Operation

**Published:** 1868-09

**Authors:** J. F. Miner


BUFFALO

### Medical and Surgical Journal.

VOL. VIII. SEPTEMBER, 1868. No. 2.

### Original Communications.

ART. I.—*The Causes of Congenital Club-foot, with remarks upon the time for Operation.* BY J. F. MINER, M. D.

In my recent investigations upon the special surgery of deformities, I have collected some specimens and obtained some facts, which I hope may interest members of the profession at all curious in such matters; and am induced to write a hasty description of some of them on account of a practical value which I think they possess. In speaking of congenital club-foot, almost all authors embody the sentiment, that its etiology has never yet been satisfactorily explained. The various theories concerning its causes are enumerated and the fallacy of all pointed out. Prof. Gross seems wholly unsettled in his opinions, and shows his indecision in the following paragraph: "The hypothesis of arrested development so warmly advocated by some modern pathologists, is altogether untenable, being essentially contrary to the facts of the case in every particular. The imperfect growth, if any such really exist, is not congenital, as this doctrine teaches, but acquired, being the result of causes which are brought to bear upon the child during its intra-uterine life, leading to shortening and contraction of certain muscles, and not to a want of development properly so-called. It must be acknowledged, however, that instances occasionally occur, although rarely, which strongly favor the doctrine under consideration. Thus I have, in my own practice, seen two infants born at the full term, but who died immediately after birth, who had each well marked harelip, cleft-palate, and club-foot, the result evidently, so far at least, as we can judge of such an occurrence, of an arrest of development." This statement is not very clear, and is a mixture of language or mixture of ideas, or both. Arrest of development, he says, is untenable, being contrary to facts in every particular. He then relates his own observation in two cases, and says, the club-foot, harelip, and cleft-palate, were evidently, so far, at least, as we can judge of such an occurrence, the result of an arrest of development. It will be observed that cleft-palate and harelip are classed with, and apparently supposed to be from the same cause as club-foot; this supposition is probably groundless. At least there is a good way of accounting for non-union of the two sides, which in no way can be applied to club-foot; at the same time, it must be acknowledged that club-foot is often associated in the same subject with other malformations and sometimes with deficiencies. If toes are left off from an otherwise good foot, it is certainly an arrest of development, but there is no arrest in club-foot, it is only misshapen. Every part of a natural foot is found in congenital club-foot, nothing is wanting, it is fully developed. Prof. Gross' two cases show nothing for the theory of arrested development, so far as club-foot is concerned.

Following the same author, as his views are fair representatives of all, or nearly all, excepting those who have proposed the various theories and advocated them to the exclusion of all others, we find the following discussion: "Another hypothesis of the formation of club-foot that has met with considerable notoriety, is, that the distortion is caused by the pressure of the uterus upon the feet of the infant during gestation, in consequence of a deficiency of the amniotic fluid. But the question may be asked, if such an effect may be exerted by this organ upon the feet, why should it not be exerted also upon the hands, head, nose, chin, legs and knees? Such a coincidence, suppose the doctrine to be true, ought to be of constant occurrence, yet it is so rare that it is probably not noticed once in a hundred cases of the affection. Besides, it remains to be proved that women who bear club-footed children have always a deficiency of amniotic fluid." He then goes on to say, "The most plausible view, perhaps, that can be framed, in the present state of the science, of the formation of club-foot, is, that it is produced by a defect of nervous influence, leading to a permanent contraction of certain muscles and a corresponding retraction and incurvation of the bones into which these muscles are inserted." It is very difficult to see any defect or deficiency of nervous supply in congenital club-foot; the muscles, tendons and ligaments are well adapted to the deformed foot, and left as it is found, it may be perfect as any foot, but in bad shape. It is true that in some cases of club-foot the whole muscular apparatus of the leg suffers—is atrophied and weakened, and this can be explained quite satisfactorily. There is no intention to disprove anything which has been proposed; the object is rather to show what is the frequent cause, and allow the existing theories to remain as far as possible undisturbed

The first case which attracted my attention was the product of an abortion at about the fourth month. The fœtus and placenta were expelled in a mass, having apparently been separated from the uterus for some days. Upon rupturing the membranes the legs were found flexed closely upon the anterior surface of the body, the feet extending as high up as the top of the head. One foot was pressed closely upon the forehead, making a deformity of the face and head as well as the foot. The foot has no form of a natural one, but is flattened completely, and fitted to the position where it grew. The other leg is likewise fitted to the side of the trunk and bent around the shoulder, fitting closely. The feet cannot be extended without rupturing the flexor muscles and tendons of the thigh.

The causes of premature expulsion are uncertain, but my conjecture is that so solid and unyielding a mass, unsurrounded by the usual amount of amniotic fluid, could not be tolerated by the uterus, and that the cause of expulsion was the accidental (if I may so speak) absence of this liquor. But I will not venture very far into the regions of conjecture, but confine myself to simple facts. Whoever examines this specimen will certainly be convinced that the deformities in this case are due to the circumstances of growth, and that there is no doubt about it, the evidence is too positive to admit of any doubt; nothing but this could possibly have plastered or spread the legs and feet out over the body in such manner as here observed. One hand is also reflected back upon the forearm and as obviously deformed as the feet. So far as any one case can settle the point it is done in this instance,—legs, feet and one hand are malformed, and there is a deep depression upon the face and forehead, where the foot rested. That hands are not as often malformed by pressure of the uterus as the feet, is easily understood; they are differently situated, less exposed to pressure when there is absence of amniotic fluid. Probably this is the only reason that we so rarely observe mal-positions of the hand—they are not at either extremity, but in the centre and protected. If deformity was due to a "defect of nervous influence," the hand and arm would as often suffer as the foot or leg.

A second instance, illustrating the same point, was that of a little boy whose case I have formerly reported as showing the benefits of tenotomy in correcting the most fearful deformities. His legs and feet had grown flexed upon the anterior portion of the trunk, so that he was a wad of a boy, with no power of extending his lower extremities until nearly all the flexor tendons of both the thighs, legs and feet had been divided. He was a living counterpart of the fœtus I have described. There was no nervous deficiency or arrested development; when the tendons had been divided and the lower extremities extended the nervous and muscular power was normal, and the former little mass of deformity now walks upright and is quite a specimen of humanity.

Prof. Rochester, not many months since, directed a little child to me for correction of deformity, or more properly, that I might see how fearfully deformed a child might be born. The thigh, knee and ankle-joints were all deformed, but that which I wish to mention in this connection as showing the *cause* of the deformity is this, a deep sulcus was observed at about the middle portion of the back part of each thigh, the muscles were divided as if nature had been attempting an amputation, in a somewhat awkward way. The midwife in attendance at the birth, said that the umbilical cord had passed around the body and inclosed the legs, fastening them closely to the body. This might have been known and would have been seen at a glance, if the thighs had been placed as they evidently grew in the uterus. The suggestion once made, it was obviously true and explained much of the existing deformity.

Dr. M. H. Shaw presented me an acephalus child born at full term, the placenta and child being expelled with membranes unruptured. I preserved the specimen as it was, placenta, membranes and child, as there appeared to be no amniotic liquor. Recently I have separated the child from the membraneous sac and find that it closely inclosed the child, the left leg was most exposed to pressure as the right one was drawn up a little more upon the abdomen. I found club-foot on the left side and could place the child and sac so as to show unmistakable cause of the deformity.

I have several other specimens equally instructive on this point, any one of which could not fail to convince the most skeptical that at least in these cases, intra-uterine pressure caused the deformity. It appears very remarkable that a cause so adequate, so evident in some cases, and so manifestly liable to be in operation, should have received so little recognition, and it is still more remarkable that from so natural and philosophical an explanation physicians should wander off into occult and inconceivable influences of which they know nothing.

True club-foot is congenital—it does not occur after birth. If from arrest of nervous supply, as is common from cerebro-spinal, and other diseases, the muscles of the leg are rendered unsteady or left beyond the control of the will, and the ankle turns loosely, it constitutes a tolerable good imitation of club-foot. Some forms of talipes are better imitated in this way than others, talipes equinus, for instance, but rarely, if ever, do we observe the deformities of the bone which character true talipes acquired in this way. If the tendons are divided which draw the foot from natural postion in such cases, it regains itself quite perfectly so far as dependent upon the form of the bones. In intra-uterine growth the slightest compression is doubtless adequate to determine the shape, and mainly on this account nature has provided an element in which the fœtus is designed to be wholly protected, where the shape of every organ may be determined by natural law, floating unconstrained in the free exercise of many of the muscles of motion. To prove the deficiency of amniotic fluid in most cases of club-foot must be difficult from the very nature of things, unless we accept the deformities caused by pressure, such as I have described as proof; to my mind they are positive proof.

*It is not all important* that we settle the cause of this deformity, but it is well to entertain correct views in this respect, for upon our convictions as to its cause will depend the time and manner in which we shall advise measures for correction. On this account mainly I have indulged in quite a lengthy discussion, which otherwise would possess very little practical value. The outline of the deformed foot has been determined by pressure during a part or the whole of intra-uterine life and when the child is born, the form of the bones, the length of the tendons and ligaments, and all the parts harmonize with each other—it is a perfect deformed foot. The bones are yet soft and pliable to some extent, at least, the ligaments are also yielding, and the muscles have never learned the art of contraction by reflex action. Sensation, also, is new and untried, and from this or other causes, it seems much less acute than a few days later. We can *now* mould the foot, not as we could have done at the fourth or fifth month of uterine life, but can do it astonishingly well. If we advise delay, as is almost universally done, for six, twelve or more months, the golden opportunity is lost, and the bones become consolidated in their unnatural shape, and can never again be as perfectly restored as at the very time of birth.

It has recently been proposed to correct these deformities without the division of tendons; this is the only time when it can be done successfully. If months are allowed to pass, the tendons must be divided and the difficulty of remoulding the foot is greatly increased. The necessity of dividing the tendons is a subject which it is not proposed at present to introduce, and there are so many other topics closely connected with the operation for cure, such as the amount of success attainable and the best means of retaining the parts in position, that remarks seem incomplete if confined to the causes of club-foot and the time of operation.

